# Supplementary material for: Fragmentation patterns and personalized sequencing of cell‐free DNA in urine and plasma of glioma patients
Source: EMBO Mol Med. 2021 Jul 22;13(8):e12881. doi: 10.15252/emmm.202012881 (PMC8350897; doi:10.15252/emmm.202012881)
Supplement: Supplementary file 1 — Appendix [file EMMM-13-e12881-s009.pdf]

Appendix for:

Fragmentation patterns and personalized sequencing of cell-free DNA in urine and plasma of gliomas.

Florent Mouliere[1,2,3,@], Christopher G. Smith[1,2,@], Katrin Heider[1,2], Jing Su[1,2], Ymke van der Pol[3], Mareike Thompson[1,2], James Morris[1,2], Jonathan C.M. Wan[1,2], Dineika Chandrananda[1,2,$], James Hadfield[1,2,§], Marta Grzelak[1,2], Irena Hudecova[1,2], Dominique-Laurent Couturier[1,2], Wendy Cooper[1,2], Hui Zhao[1,2], Davina Gale[1,2], Matthew Eldridge[1,2], Colin Watts[4,£], Kevin Brindle[1,2,#], Nitzan Rosenfeld[1,2,#], Richard Mair[1,2,4,#]

Affiliations:
1. Cancer Research UK Cambridge Institute, University of Cambridge, CB2 0RE, Cambridge, UK.
2. Cancer Research UK Major Centre – Cambridge, Cancer Research UK Cambridge Institute, CB2 0RE, Cambridge, UK.
3. Amsterdam UMC, Vrije Universiteit Amsterdam, Department of Pathology, Cancer Centre Amsterdam, 1081 HV, Amsterdam, The Netherlands.
4. Division of Neurosurgery, Department of Clinical Neurosciences, University of Cambridge, CB2 0QQ, Cambridge, UK.

@ co-first authors
# co-senior authors

Corresponding authors: f.mouliere@amsterdamumc.nl; kmb1001@cam.ac.uk; nitzan.rosenfeld@cruk.cam.ac.uk; richard.mair@cruk.cam.ac.uk;

$ Current affiliation: Peter MacCallum Cancer Centre, Melbourne, Victoria 3000, Australia; Sir Peter MacCallum, Department of Oncology, The University of Melbourne, Victoria 3010, Australia.
§ Current affiliation: Precision Medicine, R&D Oncology Unit, AstraZeneca, CB4 0WG, Cambridge, UK.
£ Current affiliation: Institute of Cancer Genomics Science, University of Birmingham, B15 2TT, Birmingham, UK.

Table of contents:

Appendix Figure S1: quality control assessment of the WES data from the tumor tissue DNA.

Appendix Figure S2: mutation class depending on the tissue or body fluid sequenced.

Appendix Figure S3: quality control and background sequencing error rates of the capture sequencing panels for CSF, plasma and urine samples.

Appendix Figure S4: cfDNA fragmentation of urine samples determined using sWGS per individuals.

Appendix Figure S5: Influence of the age on the urine cfDNA fragmentation from healthy individuals.

Appendix Figure S6: Selecting “peaks” and “valleys” for the determination of the 10 bp oscillations amplitude.

Appendix Figure S7: Clustering of cfDNA fragmentation features recovered from sWGS using 10bp binning.

Appendix Figure S8: Evaluation of the fragmentation features determined from sWGS of urine samples using the 30 bp binning.

Appendix Figure S1:

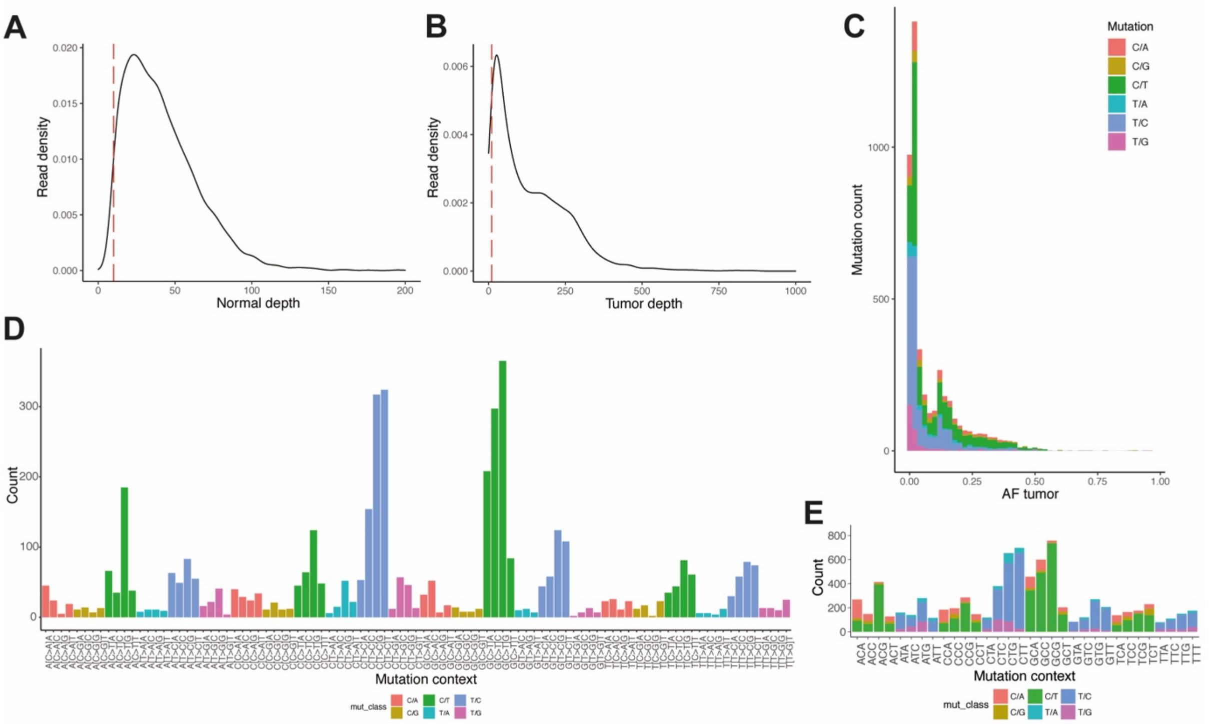

Appendix Figure S1: quality control assessment of the WES data from the tumor tissue DNA.

A: Sequencing depth from white blood cell DNA extracted from the buffy coat layer. **B:** Sequencing depth from the tumor tissue DNA. **C:** Mutation counts depending on the AF, colored by mutation class. **D:** Mutation context of the tumor tissue DNA data. Are displayed the unique patient-specific polished filtered data retained by the INVAR algorithm. **E:** Mutation counts by trinucleotide context, colored by mutation class.

Appendix Figure S2:

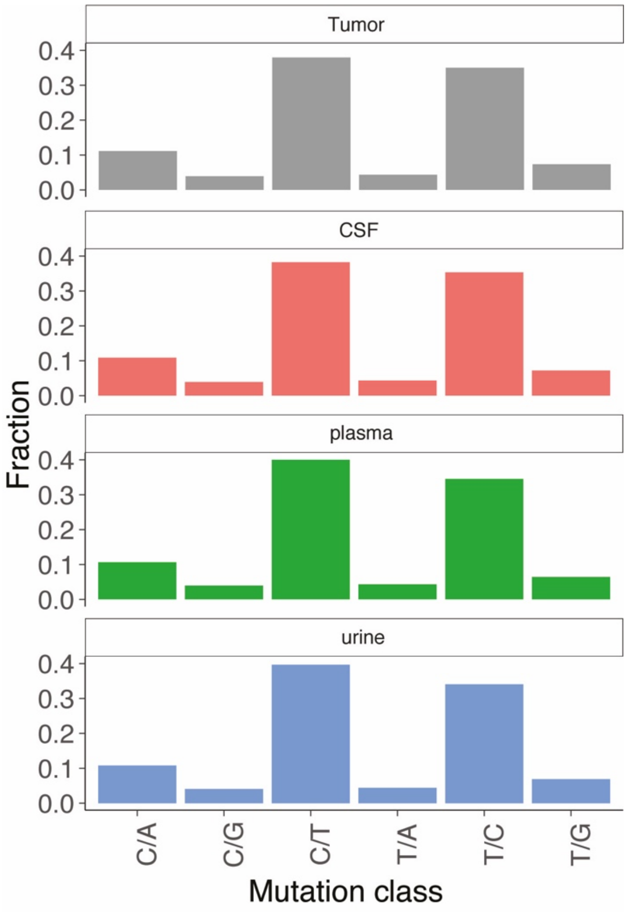

Appendix Figure S2: mutation class depending on the tissue or body fluid sequenced.

In grey are displayed tumor samples, in red are displayed CSF samples, in green plasma samples and in blue urine samples.

Appendix Figure S3:

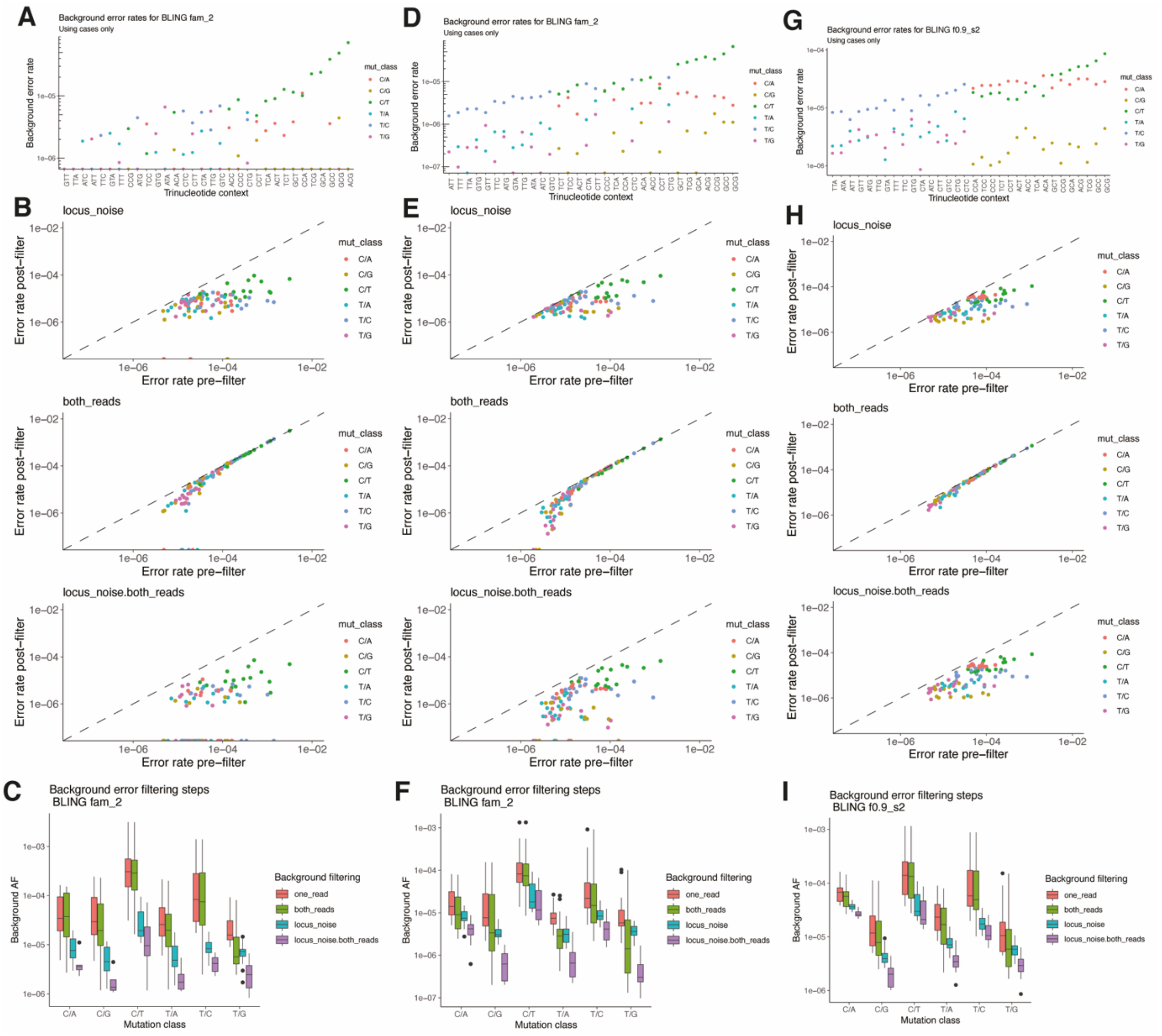

Appendix Figure S3: quality control and background sequencing error rates of the capture sequencing panels for CSF, plasma and urine samples.

A: Background error rates per mutation class for CSF samples. **B:** Background error rates per trinucleotide are plotted before and after each background error filtering steps for each trinucleotide context for CSF samples. **C:** Summary of the error rate per class depending on the filtering steps for CSF samples. **D:** Background error rates per mutation class for plasma samples. **E:** Background error rates per trinucleotide are plotted before and after each background error filtering steps for each trinucleotide context for plasma samples. **F:** Summary of the error rate per class depending on the filtering steps for plasma samples. **G:** Background error rates per mutation class for urine samples. **H:** Background error rates per trinucleotide are plotted before and after each background error filtering steps for each trinucleotide context for urine samples. **I:** Summary of the error rate per class depending on the filtering steps for urine samples.

Appendix Figure S4:

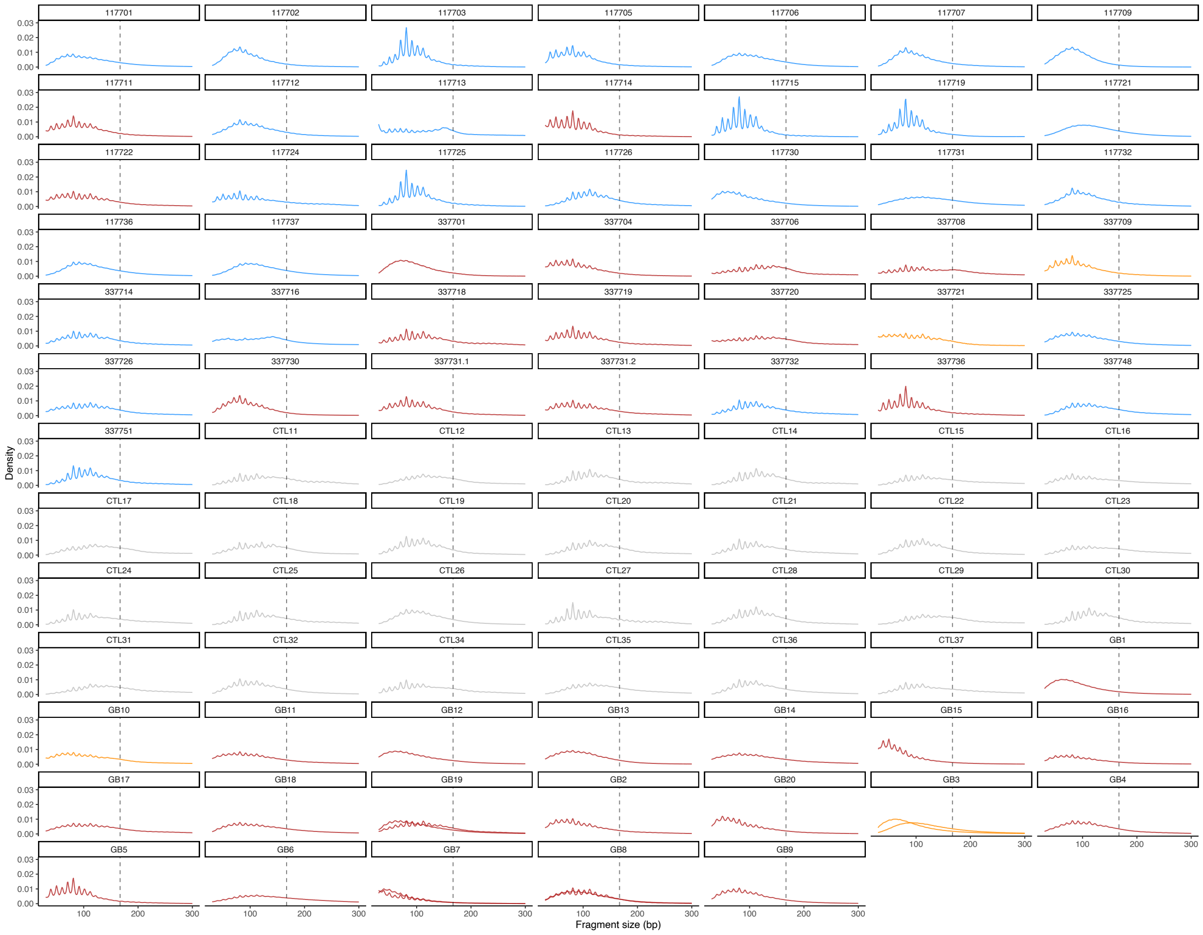

Appendix Figure S4: cfDNA fragmentation of urine samples determined using sWGS per individuals.

The colors indicate HGG (in red), LGG (in orange), healthy cases (in grey) and other CNS diseases (in blue).

Appendix Figure S5:

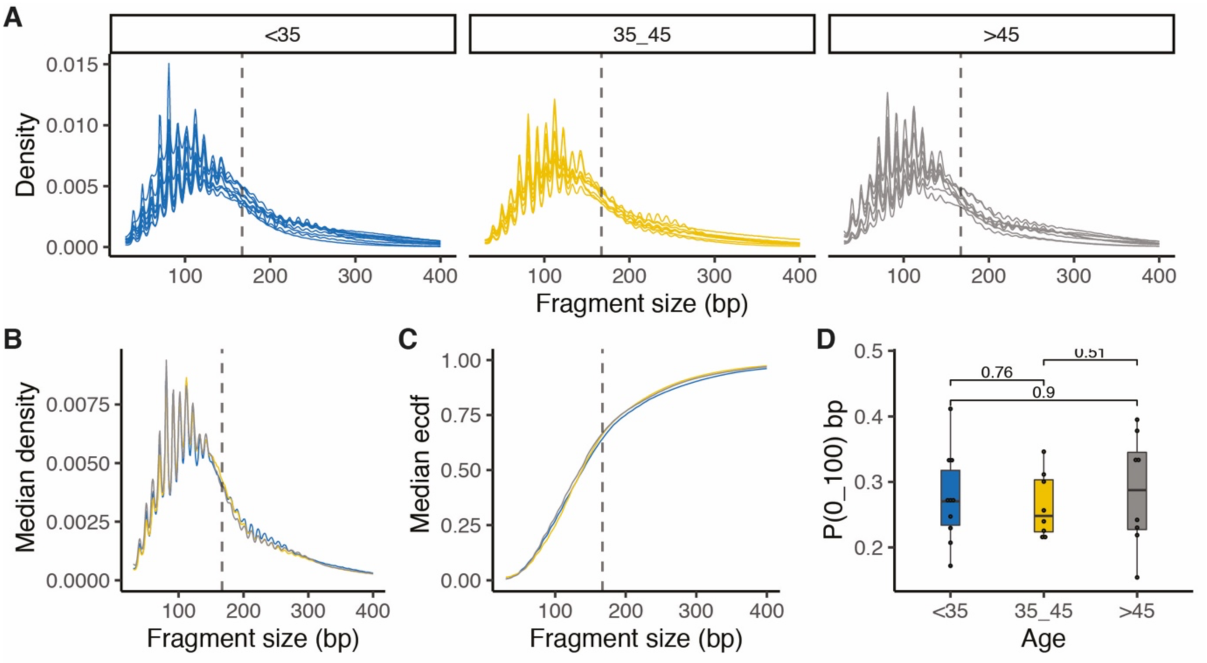

Appendix Figure S5: Influence of the age on the urine cfDNA fragmentation from healthy individuals.

Colors represent individuals <35 years old (n=8, blue), between 35 and 45 years old (n=9, yellow), and >45 years old (n=8, grey). Age is unknown for 1 individual (not shown). The median age of the healthy individuals is 41 years old (range: 23-61). **A:** cfDNA size distribution per age group. **B:** median cfDNA size distribution. **C:** median empirical cumulative distribution (ecdf). KS-test showed no significant difference. **D:** proportion of cfDNA fragments <100bp depending on the age group. No significant difference between the group can be detected (Wilcoxon-test).

Appendix Figure S6:

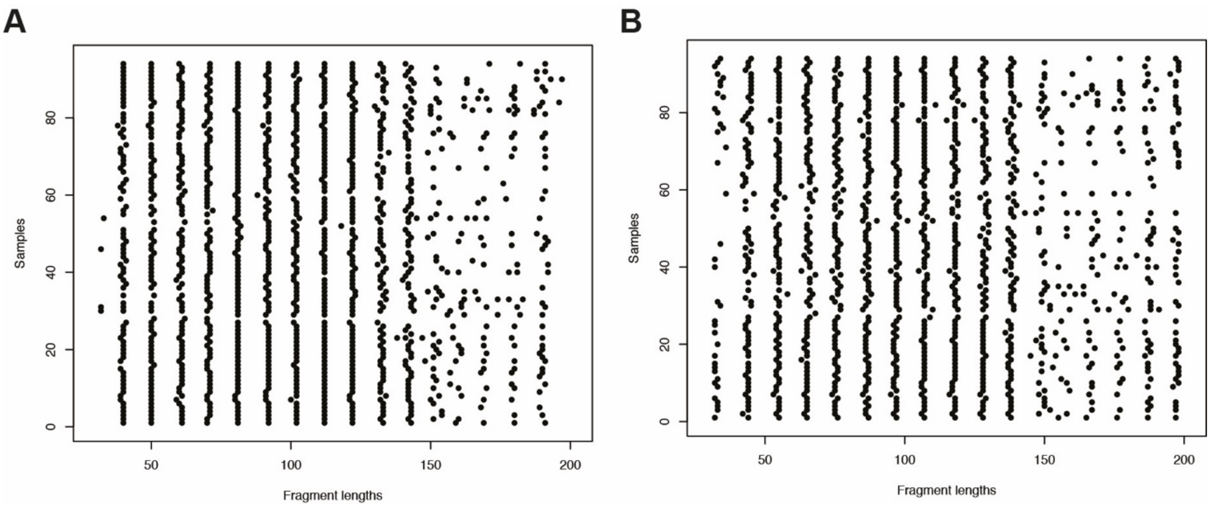

Appendix Figure S6: Selecting "peaks" and "valleys" for the determination of the 10 bp oscillations amplitude.

Each dot represents the local maxima ("peak") and local minima ("valley") depending on cfDNA fragment size determined from the sWGS of the urine samples (see Methods). **A:** local "peak" depending on the fragment length. **B:** local "valley" depending on the fragment length. The value of "peaks" and "valleys" are conserved across samples in the size range 50-140 (corresponding to a sub-nucleosome structure), but not for smaller or larger values.

Appendix Figure S7:

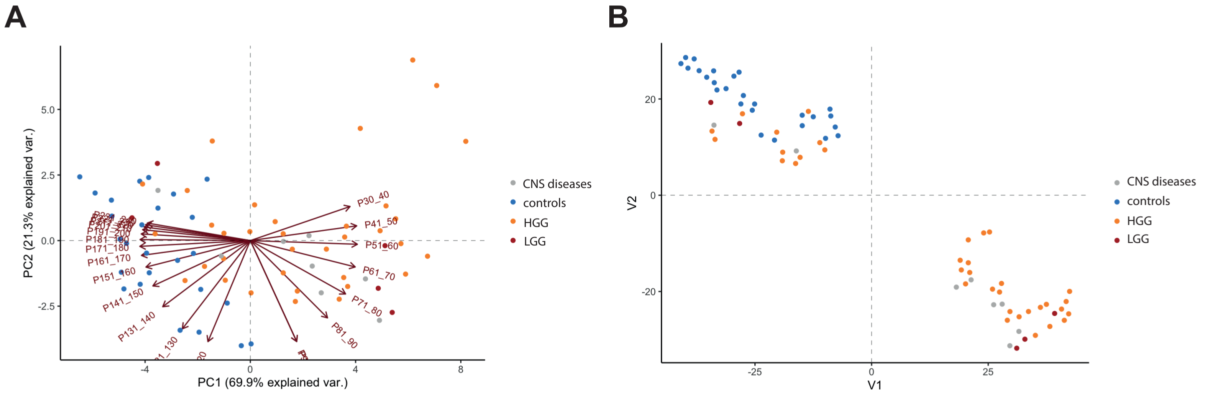

Appendix Figure S7: Clustering of cfDNA fragmentation features recovered from sWGS using 10bp binning.

A: Principal component analysis comparing cancer (HGG and LGG) and control samples (healthy and other CNS diseases) using data from the urine fragmentation features. Red arrows indicate features tested during the predictive analysis. Fragmentation features were calculated from the cfDNA fragments size (The proportion of cfDNA fragments was calculated every 10 bp bins between 30 and 300 bp); and the amplitude of the 10bp oscillations: OSC_10bp). **B:** tSNE analysis comparing cancer and control samples using data from the same urine fragmentation features.

Appendix Figure S8:

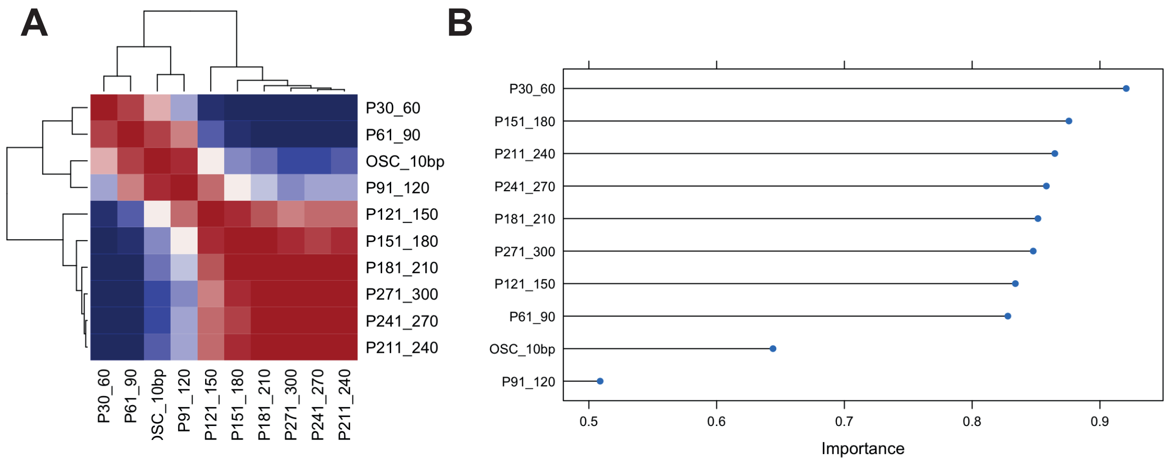

Appendix Figure S8: Evaluation of the fragmentation features determined from sWGS of urine samples using the 30 bp binning. A: Correlation matrix of the 10 fragmentation features determined by sWGS from the 74 urine samples included in the training and validation dataset of the classifier models. The correlation score was estimated for each cross-comparison, and the value displayed on as a color intensity (red = 1, blue = -1). **B:** Ranking of the individual features importance calculated with a Learning Vector Quantization (LVQ) model.
